# What about the Cytoskeletal and Related Proteins of Tapeworms in the Host’s Immune Response? An Integrative Overview

**DOI:** 10.3390/pathogens12060840

**Published:** 2023-06-18

**Authors:** Diana G. Ríos-Valencia, Javier Ambrosio, Rocío Tirado-Mendoza, Julio César Carrero, Juan Pedro Laclette

**Affiliations:** 1 Department of Microbiology and Parasitology, School of Medicine, Universidad Nacional Autónoma de México, Coyoacán, Ciudad de México 04510, Mexico; riosdg@iibiomedicas.unam.mx (D.G.R.-V.); rtirado@facmed.unam.mx (R.T.-M.); 2 Department of Immunology, Biomedical Research Institute, Universidad Nacional Autónoma de México, Coyoacán, Ciudad de México 04510, Mexico

**Keywords:** Cestoda, tapeworm, cytoskeleton, diagnosis, vaccine, drug targets

## Abstract

Recent advances have increased our understanding of the molecular machinery in the cytoskeleton of mammalian cells, in contrast to the case of tapeworm parasites, where cytoskeleton remains poorly characterized. The pertinence of a better knowledge of the tapeworm cytoskeleton is linked to the medical importance of these parasitic diseases in humans and animal stock. Moreover, its study could offer new possibilities for the development of more effective anti-parasitic drugs, as well as better strategies for their surveillance, prevention, and control. In the present review, we compile the results of recent experiments on the cytoskeleton of these parasites and analyze how these novel findings might trigger the development of new drugs or the redesign of those currently used in addition to supporting their use as biomarkers in cutting-edge diagnostic tests.

## 1. Introduction

Cestodes are invertebrate parasitic flatworms usually requiring one or two hosts for their transmission [1]. Tapeworms are a subgroup of highly specialized parasites causing zoonotic diseases of economic importance and human infections that still constitute a public health problem in developing countries of Africa, Southeast Asia and the Americas [2,3]. These parasitoses are a growing concern in developed countries receiving migrants from endemic areas [4]. The study of tapeworms has been approached from several disciplines such as parasitology, immunology, cellular biology and physiology for several reasons: 1. They are evolutionarily ancient organisms lacking a digestive tract, absorbing nutrients and discarding excretion/secretion (E/S) products through a syncytial tegument covering the entire body [5]; 2. They have evolved life cycles involving several developmental stages in different hosts (intermediate and definitive), 3. They are complex organisms able to modulate or disguise the host’s immune system in order to survive for long periods in the presence of an immune response [6,7]. Usually, the larvae and adult stages are involved with host-causing diseases in humans and livestock [8].

### Tapeworm Overview

Tapeworms are a group of parasitic organisms (Cestoda) comprising about 5000 species belonging to the phylum Platyhelminthes, with more than 30,000 species described [9]. Other clades of parasitic flatworms are Monogenea and Digenea, defined by the number of hosts required to complete their life cycles. Class Cestoda is divided in two subclasses: Cestodaria and Eucestoda [10,11].

As for tapeworms, the embryonated egg stage can develop into different larval forms called plerocercoid, cysticercoid or cysticercus [12]. The larval stage, also called metacestode, includes the metamorphosis of the oncosphere to the first evidence of sexual differentiation that results in the adult worm. In general, metacestodes can be vesiculated or pseudovesiculated, with a tissue wall limiting the fluid-filled vesicle. In the vesicular larvae, the scolex is invaginated and introverted [13]. Adult tapeworms are intestinal parasites in vertebrates, with biological cycles requiring a vertebrate or invertebrate intermediate host in which the larval form develops. The worm is ribbon-shaped and dorso-ventrally compressed, with a body organized in three regions: scolex, neck and strobila [14]. Strobila is the evolutionary result of a segmentation process producing the multiplication of the reproductive apparatus. In tapeworms, the scolex is considered the anterior region, whereas the strobila is considered posterior [8,15]. To survive in the gut or the tissues of their intermediate or definitive hosts, tapeworms maintain a continuous exchange of molecules with their hosts through a syncytial layer known as the tegument. The external surface of the tegument is greatly increased through highly structured membrane folds known as microtriches [16]. It is also worth emphasizing that tapeworms lack a digestive tract; therefore, all exchange with their hosts happens through the tegument.

The tegument’s function is supported by underlying cell types, including muscle, glycogen, and fat storage cells, as well as other structures related to energy production [16], waste elimination, neural transmission [17,18]. In many cases, the whole tissue organization is syncytial. Among the cell types present in tapeworms are flame and excretory canal cells, smooth muscle, fibroblasts, and neurons [19,20]. The specific function of calcareous corpuscles is related to the trapping of waste compounds and osmoregulation; recently, it has been discovered that calcareous corpuscles are involved in the uptake and disposal of host proteins [21]. The muscular system of most tapeworms is composed of smooth muscle cells and is made of highly organized muscle layers [22]. These layers allow the motility shown by the tapeworm since the adult worm can actively crawl and the larvae show synchronized sphincter-like movement [21].

There is a wide variety of tapeworms of medical and veterinary importance, including *Taenia solium* and *T. asiatica*, which cause human and porcine cysticercosis [2,23,24]; *Taenia crassiceps* has been extensively used as an animal model for human cysticercosis [25,26]; *Echinococcus granulosus*, also included in the Taenidae family, is the causal agent of human hydatidosis; *E. multilocularis* also produces small alveolar cysts (causing a multilocular infection) that can metastasize to different organs. Members of the Hymenolepididae family such as *Hymenolepis nana* and *H. diminuta* mainly affect rodents, but man is also considered a regular host for these parasites [27].

Finally, most species of tapeworms are hermaphroditic; in the adult segmented worm, each segment develops male and female gonads. In some cases, self-fertilization is a common trait [28]. This group of organisms originated more than 600 million years ago and are exquisitely adapted to a parasitic way of life, possessing highly simplified genomes characterized by a lack of biosynthetic metabolic abilities [29,30].

The first aim of this review is to assemble recent information available on the cytoskeleton of tapeworm parasites, including that which is related to immune recognition and involvement in the host–parasite relationship; the second aim is analyzing the potential of these findings on development of new drug treatments, as well as diagnostic and vaccine candidate molecules.

## 2. Cytoskeletal Proteins Identified in Tapeworms

### 2.1. Microfilaments and Muscular System

**Figure 1:**
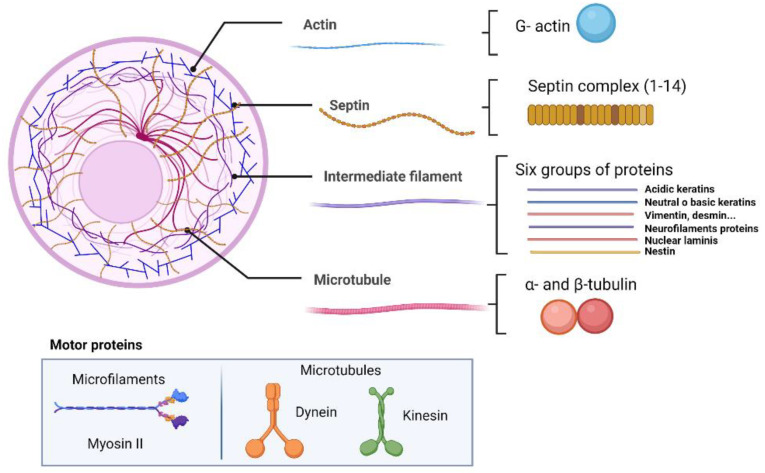
Architecture of cytoskeleton.

Actin and its associated motor proteins (myosin, tropomyosin and paramyosin) in tapeworms are involved in mobility and dynamic functions, as well as in the maintenance of the shape and size of the parasites (Figure 1). Microfilaments are part of the tapeworm contractile machinery, being widely distributed in the tegument and muscle fibers located in the syncytial parenchyma, as well as in flame cells [31,32,33,34].

#### 2.1.1. Actin

Actin is a multifaceted protein with a molecular weight of around 41–42 kDa (Figure 1). It has been established that all eukaryotes have one or more genes for actin; sequence comparisons have showed that actins are among the most conserved gene families [35,36], which participate in functions such as muscle contraction, cell mobility and division, cytokinesis and organelle movement, cell signaling, and the establishment and maintenance of cell junctions and shape. Many of these processes are mediated by extensive and intimate interactions of actin with cell membranes [37]. Actin was the first tapeworm gene cloned and sequenced [38], being found as a highly expressed protein [29,34]. In the *E. granulosus* scolex secretome 44 antigenic proteins were identified; actin was identified as one of the most abundant. Therefore, actin has been proposed as a diagnostic marker or pharmacological target for equinococosis [39].

**Figure 2:**
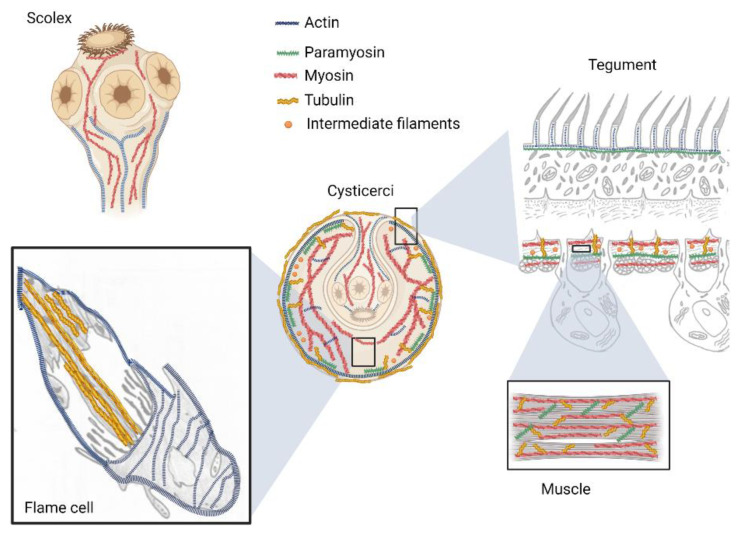
Main locations of cytoskeleton proteins in tapeworms.

Studies have shown the presence of seven actin isoforms in *T. solium* [40] and six in *E. granulosus* [41]. The protein has been identified mainly in the tegument and in the protenephridial system, specifically in the terminal part of flame cells (Figure 2) [21,33]. In *D. dendriticum*, the study of actin expression in different developmental stages of the parasite, showed that filaments of this protein are present in all stages of the life cycle [42]. In *M. corti*, actin was in the main muscle layers (outer circular and inner longitudinal), as well as in the suckers and flame cells [43]. On the other hand, the search for actin in *T. crassiceps* by confocal microscopy and TEM demonstrated its presence in the rings surrounding the cilia plumes of the flame cells. Actin was detected as a globular protein in the microvilli of the tegument, muscle fibers and rings surrounding the cilia of the flame cells [44]. In *E. granulosus*, actin cytoskeleton is considered among the most important proteins for muscle contraction involved in the invagination/evagination process [41].

#### 2.1.2. Myosin and Paramyosin

One of main motor proteins in muscle is myosin II, capable of binding F-actin and hydrolyzing ATP and participating in a wide variety of processes such as muscle contraction and cell migration (Figure 1). The structure of myosin fibers consists of two heavy chains, two light chains and two regulatory light chains, working by polymerization into extended filaments [44,45].

Immunohistochemical studies in tapeworms have shown that myosin is mainly expressed in the bladder wall and the spiral canal of *T. solium* and *T. crassiceps* cysticerci (Figure 2) [34,46]. In the adult worm, myosin was localized in the suckers and the neck (with long filaments that connect the scolex with the strobilus) as well as in the proglottids. The use of home-made specific antibodies against *T. solium* myosin (not commercially available) allowed identification of four isoforms which appear to be related to physiological requirements of each parasitic stages [34,46].

Paramyosin is a protein with a MW of 98–105 kDa, characteristic of invertebrates [47], including tapeworms that are parasites of humans and domestic animals, such as *T. solium*, *T. crassiceps*, *T. saginata* and *E. granulosus* [34,48,49,50]. Paramyosin is known to be part of the thick filaments together with myosin. This protein forms homodimers with supercoiled α-helix secondary structure, located in the central part of the filaments, with two myosin molecules around this paramyosin nucleus (Figure 2). Paramyosin has the unique ability of allowing contracture of skeletal fibers without expenditure of energy [47,51].

Paramyosin was originally identified as a prominent antigen formerly called antigen B in *T. solium* (AgB) and is one of the best characterized proteins of cysticerci. This protein, found in muscle and tegumental structures [52] has the property to bind C1q resulting in a blocking of the complement cascade. Since the complement plays a role in the modulation of the host inflammatory response, paramyosin was involved on the reduced inflammatory infiltrate observed around cysticerci in the host tissues and the maintenance of homeostasis in the host–parasite relationship [48,53]. Three isoforms of paramyosin have been proposed to occur in *T. crassiceps*, located in the distal muscle fibers and in some muscle fibers of the interstitial matrix, but not in the flame cells [34]. Paramyosin and other cytoskeleton proteins were found in two stages of *H. diminuta* by an immunoproteomic approach [53,54,55].

#### 2.1.3. Tropomyosin

Tropomyosins are a family of actin-binding proteins that are important in both muscle and other cells [56]. Their structure is composed of alpha-helical proteins that bind to the muscular and non-muscular actin filaments. In the muscle, tropomyosin mediates contraction through the regulation of actin-myosin, and is involved together with troponin, in regulating the interaction of actin/myosin thin and thick filaments during muscle contraction [57,58]. At least two isoforms of high (HMW) and low molecular weight (LMW) tropomyosins have been reported [56,59].

Two different HMW tropomyosin isoforms of 43 and 38 kDa have been identified in *M. corti* by 2D electrophoresis gels using mass spectrometry [43]. The expression of these proteins was studied during the *M. corti* segmentation process using a polyclonal antibody generated against an HMW tropomyosin of *E. granulosus*. These results showed that HMW tropomyosins are mainly expressed in muscle layers for development of muscle fibers in the genital ducts [43]. In *E. granulosus*, expression analysis of tropomyosins by immunorecognition allowed identification of three bands in total protein extracts of protoscolices and suction cups; detection of another weak signal could correspond to non-muscular tropomyosin isoforms [60].

### 2.2. Microtubules

Microtubules, considered essential to eukaryotic cells, are composed of groups of filaments composed of heteropolymers of α-tubulin and β- tubulin proteins and their motor proteins dynein and kinesin (Figure 1). In eukaryotes, the microtubules are mainly located in the primary structural components of the mitotic spindle, in the mammalian midbody, in the flagellar axonemes, and in the cytoplasmic arrays used for vesicle trafficking. As such, microtubules are important in many different processes in cells, such as the maintenance of cell structure, cell transport, protein trafficking, mitotic spindle organization, chromosomal segregation, and cytokinesis [61,62]. All these functions are performed by the interaction of microtubules with other proteins known as microtubule-associated proteins (MAPs) and with other tubulin isotypes. Moreover, tubulins can undergo posttranslational modifications such as tyrosination/detyrosination, Δ2-tubulin formation, acetylation, phosphorylation, ubiquitination, glutamylation, and glycylation that are important in performing certain functions of microtubules [63]. In tapeworms, microtubules have been studied and characterized; tubulins are normally found in the genome, proteome, transcriptome, and surfaceome of some tapeworms [29,54,64].

#### 2.2.1. Tubulin

Tubulin is a 50 to 55 kDa protein expressed in different isotypes by specific genes with spatially and temporally regulated expression levels [65,66]. The most important families are the α and β tubulins that polymerize into protofilaments during the cell cycle to form microtubules [67]. The α and β tubulin monomers differ in their amino acid sequence and are encoded by different genes [66]. Tubulins have been localized in the tegument and the ciliary tufts of flame cells from *T. solium* and *T. crassiceps* tapeworms [34,40]. The localization of tubulin in the syncytial layer have been related to the requirement of tapeworms to maintain the exchange of substances through and within the syncytium using vesicular trafficking (Figure 2). The association between actin and α-tubulin in the tegument of these parasites has been reported and has been related to muscle activity and movement [34].

As mentioned above, α-tubulins can undergo a wide variety of post-translational modifications such as tyrosination/detyrosination, phosphorylation, glutamylation, and acetylation; modifications that have been implicated in specific functions in tapeworms [68]. Thus, acetylated α-tubulin observed in the cytoplasm and cilia of flame cell in the collecting ducts of *H. diminuta*, and in other ciliated structures, as well as in the tegument region called sensilla, have been shown to participate in chemosensitivity and/or mechanosensitive processes [21]. In *Echinococcus*, α-tubulins have been used to study the nervous system, and its modified form was shown to have an important role in this system and is crucial for the survival of this parasite [69]. It is worth mentioning that the mechanism of action of mebendazole was initially identified from in vitro tubulin polymerization assays [70]. Furthermore, tubulin is considered a good vaccine candidate and a good target for the design of new compounds against tapeworm infections [29,71].

#### 2.2.2. Dyneins

Dyneins are one of the three families of cytoskeleton motor proteins (Figure 1) involved in intracellular motility of vesicles and organelles along microtubules [72]. The genomes of metazoans encode five subunits of dyneins, each one forming dimers: the intermediate chain, the light-medium chain, and three types of light chain [72]. The dynein light chain has been identified by mass spectrometry in *T. crassiceps* [34] and *E. granulosus* tapeworms, and in the latter, it was also identified as a protein of the excretion/secretion system [39]. More dynein light chain (DLC) and dynein heavy chain (DHC) family members have been identified in *E. granulosus* and schistosome worms in comparison to nematodes [73].

The analysis of tegumental proteins in *Echinococcus* showed the presence of several proteins with a dynein light chain-like domain, some of them involved in ion uptake and immune evasion. In the genome of *E. granulosus* around 48 dynein light chain members were identified, this number is higher than those found in other helminths such as *S. mansoni* and *S. japonicum* [74,75]. In *S. japonicum* 20 proteins corresponding to at least 5 kinds of dynein light chain proteins have been identified whereas dynein has been documented as a tegumental antigen in *C. sinensis* [76,77,78]. The above is important if we consider that the tegument is involved in nutrition, immune evasion and modulation, excretion, signal transduction and parasite–host interface [5,79,80]. As dyneins participate in the regulation of microtubule dynamics, they have been involved with intense vesicular trafficking and ciliary movement [79,81,82], as well as with other vital functions related with secretion and elimination of toxic substances in *T. crassiceps* cysticerci [71,83].

### 2.3. Intermediate Filaments

The intermediate filament (IF) protein family is composed of approximately 70 members classified into six groups based on sequence homology, gene organization, net charge, assembly mechanism, and expression pattern (Figure 1). Some of their representative proteins are vimentin, keratins, desmins, neurofilaments, glial fibrillary acidic protein (GFAP) and a small number of minor subgroups as nestins and peripherins [84,85,86,87,88].

The IF is perhaps the less known cytoskeleton protein filament group in cestodes; however, the buffering of mechanical stress is thought to be the major function of these filaments in metazoan cells [89,90,91]. So far, some IF proteins such as vimentins, desmines, and keratins have been identified in cysticerci of *T. crassiceps* located in the body wall, subtegumental tissue, suckers, and rostellum [92]. Moreover, keratin-like proteins have been found in the embryophore of *E. multilocularis* eggs and other tapeworms [93]. On the other hand, laminin binding proteins have been characterized for *E. granulosus* being found in the cytoplasm of subtegumental cytons and myocytons; these proteins appear to play a role in the host–parasite interaction and in the establishment of the oncosphere in the host tissues [94,95].

### 2.4. Septins

Septins are the fourth filament of importance in the cytoskeleton. These are 30-65 KDa proteins with domains having GTPase activity, similar to myosins and kinesins [96]. Septins can form highly structural multimers (Figure 1), which participate with other cytoskeletal proteins in specific functions such as cytokinesis, exocytosis, and membrane remodeling [97]. Moreover, it has been shown that septins act as scaffolds for the recruitment of other proteins in yeast and metazoans [98,99,100]. Septins have been studied in *T. crassiceps* and in *T. solium* tapeworms. They participate in parasite survival when specific inhibitors (Forchlorfenuron) are used, decreasing the mobility of cysticerci and altering their morphology [101]. Similar results were also reported for the trematode *S. mansoni* [102]. In addition, genes encoding these proteins have been found in the genomes of other tapeworms [103] (Table 1). Tapeworms such as *T. solium*, *E. granulosus,* and *E. multilocularis* have two genes that encode septin 7, one for septin 10 and one for septin 4 [101,103]. However, ulterior analyses will be useful to understand the role of septins in these parasites.

### 2.5. Other Cytoskeleton Proteins

Some other cytoskeleton-related proteins have been identified in protein extracts of cestodes by mass spectrometry. Thus, gelsolin, a protein that participates in the assembly and disassembly of actin filaments increasing mobility [110], was identified in *T. solium* cysticerci [111], and severin, a protein that participates in the dissociation of the actin filaments was identified in *E. granulosus* [112]. Gelsolin and severin have been proposed to be evolutionarily related proteins, as they share structural similarities besides being calcium-dependent proteins [113]. The presence of these proteins seems to be an indication of the complex level of cytoskeleton organization in tapeworms.

## 3. Expression and Function of Cytoskeletal Proteins in Tapeworms

As in most organisms, the cytoskeleton of the tapeworms is critical in structural roles, but it is also vital for the conservation of the parasite physiology and development and therefore for their survival and infectivity. Accordingly, the search for changes in the expression of cytoskeletal proteins or their post-translational variants along different stages of cestode development, could allow determining their role in many aspects of host–parasite relationship, aiding identification of potential targets for the control of diseases they cause, as well as to contribute to the development of more efficient diagnostic methods [114].

Many genes presumably codifying cytoskeletal proteins can be retrieved from the tapeworm genomes listed in the WormBase ParaSite [115,116] and the recent genome of [30]. From the study of these genomes, it has been found that several tapeworms have numerous possible gene isoforms for specific proteins. A compendium of gene isovariants encoding for cytoskeletal proteins annotated and not-annotated in tapeworms is shown in Table 1. Some of these genes are part of a multifamily of genes that could encode many variants of the same protein. Such is the case of dynein, a protein encoded by 72–75 genes in *E. granulosus* and *E. multilocularis* genes in contrast to only one or two genes encoding keratin and vimentin (Table 2). In general, the protein variants of the cytoskeleton are poorly characterized in tapeworms, and even the expression of some of them has not been approached.

The information obtained from the genomic, transcriptomic, and proteomic analysis of tapeworms has shown that cytoskeletal proteins with the highest levels of expression are actin, tubulin, laminin, and myosin [29,117]. In T. solium, H. diminuta and E. granulosus, several isoforms of actin, myosin II, and paramyosin are highly expressed and have been associated with adaptation to the host environment [29,34]. Of all proteins of the cytoskeleton, actin is the most evolutionary conserved and is found in great abundance [34]. In T. multiceps, transcriptomic analysis identified 30 domains/families of greater abundance; among them, cytoskeletal proteins (α and β-tubulin, dynein, filamin and actin) were abundantly represented [117]. Finally, cytoskeleton proteins actin, actin-1, β-actin, tubulin chain 2 β3, tubulin chain 1c α, tropomyosin, tropomyosin B, high molecular weight tropomyosin isoform, paramyosin and dynein light chain, were identified in the secretome of E. granulosus scolex [39].

Cytoskeleton proteins are important elements for parasite development in all life stages and may have different and specific functions at each developmental stage, as demonstrated in transcriptomic studies on M. corti [118]. In addition to the classic functions on mobility or vesicular transit of cytoskeletal proteins, they also perform other roles in specific events: asexual reproduction by budding of cysts, size changes, etc. [119]. Transcriptomic studies have also shown that cytoskeletal proteins participate in the maintenance of the host–parasite interaction, being part of E/S processes [120,121].

## 4. Diagnosis of Tapeworm Diseases and the Cytoskeletal Proteins

**Figure 3:**
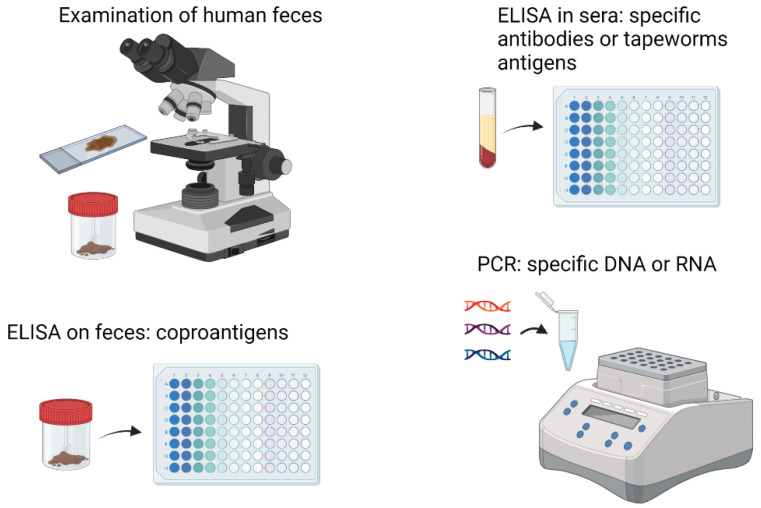
Diagnostic methods used to detect tapeworm infections.

The routine diagnosis of tapeworm infections in clinical laboratories of first-level care centers is based on the identification of proglotids and/or eggs in feces during a serial copro-parasitoscopic study (Figure 3). Egg concentration methods such as the Kato–Katz test are one of the most used for diagnosis [122,123]. In addition to these analyses, multiple diagnostic tests have been developed based on the detection of parasitic antigens, detection of antibodies raised against those antigens and detection of nucleic acids, particularly parasitic DNA. Thus, diagnosis of human taeniasis is based on a series of methods including antigen detection in feces (Copro-Ag-ELISA), immunoblot and copro-PCR (Figure 3) [12]. Serology for the detection of antigens or antibodies, either in humans or in pigs, have been implemented using protein extracts of T. solium cysts, or fractions such as cyst fluid, the bladder wall or the scolex. Assays using these preparations can reach sensitivities and specificities close to 100% [124,125,126]. Regarding T. solium cytoskeletal proteins, no studies of diagnosis based on them have been reported. However, the use of proteomic techniques such as 2D electrophoresis and mass spectrometry allowed the identification of seven proteins in T. solium that were exclusively recognized by antibodies presents in the serum of pigs with cysticerci, including cytoskeleton proteins tropomyosin and α and β tubulin, suggesting that they can be exploited as target antigens in serologic tests [127].

Diagnosis of E. granulosus and E. multilocularis can be approached similarly by detecting antibodies and, more frequently, parasite antigens or DNA in stool [128]. Several laboratory diagnostic methods have been described for the case of cystic echinococcosis (Figure 3), such as the detection of antibodies, antigens, and cytokines. However, more sensitive, and specific methods are missing, so a combination of several methods has been suggested (detection of antibodies and antigens) or combination of several (recombinant) antigens to improve the performance of complementary laboratory methods [129]. Proteomic analysis of E/S products from E. granulosus adult worms showed the immune identification of 21 spots in 2D polyacrylamide gels corresponding to 12 different proteins related to cytoskeleton, including actin, severin, and paramyosin [130]. Paramyosin was among the first antigens proposed in T. solium for immunodiagnosis [52,53,131].

The cestode Anoplocephala perfoliata affects horses; therefore, its diagnosis is important in the veterinary field [132]. Standard diagnostic methods include ELISA for equine sera and saliva [132,133] or PCR for the amplification of parasite-specific DNA in feces, but the diagnostic sensitivity of this method is only slightly higher than eggs count [134]. Therefore, development of commercially available tests to diagnose the E/S copro antigens of A. perfoliata is desirable [135,136]. Quantitative proteomics of A. perfoliata worms allowed identification of 509 E/S proteins. Immunoblot assays with sera from horses testing positive/negative for A. perfoliata, indicated stronger immunogenicity with several proteins, including the light chain component of dynein (DYNLL) and tubulin-specific chaperone A (TBCA) [136].

Regarding diagnosis through skeletal proteins, paramyosin has been identified as a useful antigen in Western blot studies using serum samples of patients with hydatid disease [137]. Immunoproteomics on extracts of different stages of H. diminuta showed the identification of some cytoskeleton proteins, including actin, paramyosin, tubulins, laminin, and filamin [138,139]. The identification and characterization of these cytoskeleton proteins can be useful in the search for new diagnosis methods, including markers that allow a quick and efficient diagnosis to have greater specificity. Several proteins have been used in diagnosis, vaccine development and drug treatment for tapeworms infections (Table 3).

## 5. Cytoskeletal Tapeworm Proteins in Vaccine-Development

A relevant aspect of tapeworm parasites is their ability to evade the host’s immune response through several molecular strategies: antigenic variation, molecular mimicry, and immune modulation, among many others [2]. These aspects of immune evasion in conjunction with their complex life cycles challenge the development of effective vaccines against tapeworms [144]. However, in most cases tapeworm infections confer immunity [145].

During tapeworm infections, a Th1-type cellular immune response profile predominates early, which then transitions to a Th2-type profile that predominates at the stage of cyst establishment and growth [146,147,148]. Th1-type response is characterized by the secretion of high levels of pro-inflammatory cytokines (INFγ, TNFα, IL-1β, IL-2, IL-12), whereas Th2-type response is characterized by the secretion of anti-inflammatory cytokines (IL-4, IL-6, IL-9, IL-10, IL-13, IL-25, IL-33, and TGF-β [146]. A polarized cytokine response plays an important role in some parasitic diseases where Th1- or Th2-type reactions are associated with susceptibility or resistance [145,148]. In tapeworm infection, the type of response associated with protection depends on the stage of the parasite and its location within the host. Thus, while a Th1 response is necessary for the elimination of larvae as they migrate in tissues, such a response exacerbated during neurocysticercosis may be counterproductive by concomitant damage to brain tissue. The same can occur in the immune response to Schistosoma while they are housed in the mesenteric blood plexus, where in this case, a balance between Th1 and Th2 responses is optimal to control parasite load and minimize collateral damage. On the other hand, the Th2 type response is considered necessary to have the option of expelling tapeworms from the intestine during their adult stage, by changing the physiology of the intestine to a more aggressive environment with abundant production of mucus, antimicrobial peptides, but above all, high intestinal muscle contractility [147,149].

The platyhelminth cytoskeleton protein that has attracted the most attention as a vaccine antigen is paramyosin. Paramyosin has been considered a promising candidate for vaccine development against infections by *S. japonicum*, *S. mansoni* and *C. sinensis* flukes, as well as *T. solium* and *E. granulosus* tapeworms [52,53,150,151,152]. This protein, in addition to binding to and inhibiting the host complement, is highly immunogenic, presenting a wide variety of B and T cell epitopes [131,153,154]. The ability of paramyosin to inhibit the complement cascade is considered one of the major immune evasion mechanisms of the *T. solium* cysticercus [52,131,140,153]. Therefore, in addition to participating in muscle physiology, paramyosin is a very important molecule at the host–parasite interface [155]. Specific antibodies against this protein preferentially recognize the carboxyl terminus, while the central domain and the amino terminus are poorly recognized [153,155,156]. In contrast, the cellular immune response seems to be preferentially directed towards the amino terminus of the protein as observed in in vitro proliferation assays of T lymphocytes from immunized mice, leading to antibodies masking the region that binds C1q [52]. Vaccination studies in mice against *T. crassiceps* cysticercosis using recombinant fragments of the protein showed that most of the protective epitopes of paramyosin reside in the amino-terminal fragment, which drove a Th1-type immune response that reduced parasitic load [154]. Similar results were obtained in DNA immunization assays performed by intramuscular injection of plasmid DNA encoding the paramyosin amino terminal sequence [52]. Noteworthy, these results were replicated in vaccination trials against cysticercosis by *T. solium*. Pigs immunized by intramuscular route with 1 mg of recombinant *T. solium* paramyosin showed 92.6% of protection when challenged with *T. solium* eggs, including four out five vaccinated pigs showing no viable cysts [157]. The decrease in worm burden was significantly associated to the combined humoral and cellular immunity against paramyosin.

Another tapeworm cytoskeletal protein with potential as a vaccine is the *E. granulosus* EgA31 antigen, a 66 kDa fibrous protein expressed at various stages of the parasite life cycle and with 20–30% similarity to platyhelminths paramyosin [158,159] Immunization assays using EgA31 alone or combined with EgFABP1 (fatty acid binding protein) and EgTrp (tropomyosin), significantly increased the Th1/Th2 profile in mice, resulting in high levels of cytokines and IgA and IgG1 titers [141,160]. Protection studies are awaiting completion [152]. Other Echinoccocal promising antigens includes cytoskeletal proteins EgTrp (tropomyosin) and gelsolin (antigen 8) [141].

The potential of microtubules, particularly tubulin, as targets for the development of new drugs and vaccines for the treatment of parasitic and fungal infections has also been proposed. In particular, the potential use of RNA interference against tubulin has been discussed as a strategy to inhibit the proliferation of helminthic parasites [161]. Although several successful studies of this type have been conducted with nematodes, no studies with helminths have yet been reported [161].

## 6. Cytoskeletal Proteins as Targets of Cestocidal Drugs

As mentioned above, the integrity of the cytoskeleton is critical for maintenance of the tapeworm’s life cycle and infectivity and therefore, tapeworm cytoskeletal proteins have largely been considered pharmacological targets [162]. In fact, albendazole, the drug of choice for treatment of tapeworm infections, interferes the cytoskeletal function by targeting β-tubulin which in turn inhibits microtubule polymerization. Impaired microtubule polymerization affects nutrient absorption and E/S processes, decreasing ATP production and release of proteolytic enzymes that lead to parasite death by starvation or autolysis [163,164]. A molecular analysis has revealed that the sensitivity to albendazole and other benzimidazoles varies in organisms according to their evolutionary distance [165], correlating with the presence of specific alleles of β-tubulin genes. Parasite tubulins have specific sites that are not found in host tubulins, making them ideal targets for tubulin inhibitors such as the benzimidazoles. However, mutation in the β-tubulin gene is a well-known phenomenon in worms leading to drug resistance. At least 28 mutations that confer resistance in *C. elegans* to benzimidazoles were described, which all mapped to a unique β-tubulin gene [166]. On the other hand, albendazole resistance in *Haemonchus contortus* and other helminths has been related with a F200Y single nucleotide polymorphism (SNP) in the isotype-1 β-tubulin gene [167,168]. Therefore, a strategy to develop new effective anthelmintic drugs is the use of different substituents in the benzimidazole nucleus to produce molecules with higher affinity and good antiparasitic activity. In addition to albendazole, there are several derivatives such as flubendazole, fenbendazole, and oxfendazole (metabolic product of fenbendazole) that are also used for the treatment of intestinal helminthiasis by affecting the parasite cytoskeleton. Flubendazole killed protoscolex of *E. granulosus* after incubation for 30 days, causing morphological changes such as soma shrinkage, integument blistering, rostellum disorganization, loss of hooks, and destruction of microtrichiae [142]. Other benzimidazole derivative is RCB20 (2- (trifluoromethyl) -1H-benzimidazole), a compound more soluble than albendazole, which showed to be highly in vitro and in vivo active against tapeworms *H. nana* and *T. crassiceps*. RCB20 changed the expression pattern and distribution of α-tubulin, actin F, and myosin II proteins, altering microtubules and cilia function that causes parasite’s death [71].

Another drug used for the treatment of cestode infections is praziquantel (PZQ). PZQ induces Ca^2+^ entry through the parasite tegument altering calcium ion homeostasis and producing a progressive disruption of muscle fibers in the worm, [169]. Besides PZQ activates gelsolin, a protein that fragment actin filaments [143], affecting myosin regulatory light chains and causing uncontrolled muscle contraction and parasite paralysis [143].

Overall, the studies have shown that the use of cytoskeletal proteins as drug targets is one of the most successful strategies for treating tapeworm infections [71,162]. However, the development of resistance to these drugs is a latent problem that must be addressed. The investigation of new treatment alternatives includes the design of more benzimidazole variant molecules or the identification of new potentially active compounds against the cytoskeleton of tapeworms.

## 7. Conclusions

The cytoskeleton is one of the most important systems of cell physiology. Its participation in such critical processes as cell division, cytoplasmic organization, cell shape, mobility, vesicular transport, intracellular signaling, and mechanotransduction, make its correct function an essential part for the survival of organisms. Along with the muscular system, the cytoskeleton is responsible for the extensive movement capacity of invertebrates such as worms, and especially adult tapeworms, which require adherence with the hooks of their rostellum and their suckers to the host’s intestine to colonize it and complete its life cycle. In fact, the drugs of choice for the elimination of tapeworms, and helminths in general, are all aimed at disabling the function of the cytoskeleton, paralyzing the worm, and thereby allowing its expulsion. With the massive sequencing of tapeworm genomes, many of the typical cytoskeleton proteins have been identified, including the identification of many isoforms, which reflects an expansion of genes that have to do with the vital function of this system. However, there is still much to be studied about the cytoskeleton of these organisms and their participation in many of the functions that are known in mammals and have not yet been addressed in tapeworms. In this regard, the advent of new techniques for manipulating protein expression, which are still in their infancy in helminths, such as gene transfection [170] and gene silencing through techniques such as interference RNA and CRISPR-Cas9, may help in short time to define with certainty the physiological function of the different proteins of the cytoskeleton of tapeworms, and their importance in parasite survival.

Another important aspect is the study of the cytoskeleton in the host–parasite relationship. In this sense, it has been shown that several of the proteins of these parasites are antigenic, which implies that once they are released into the extracellular medium due to damage to the parasite tissue or in the E/S products, an immune response is activated in the host with the generation of specific antibodies against components of the cytoskeleton. This potential of cytoskeletal proteins has only been partially exploited for the identification of new and more efficient molecular targets for vaccine development as well as for the generation of new biomarkers for diagnostic purposes. The wide potential of the proteins of the cytoskeleton of tapeworms for these purposes has not been fully exploited either, so there are still many studies in this regard that can be done and that would help to develop better control strategies for the diseases they cause in humans and in animals of human consumption.

## Figures and Tables

**Table 1:** Characteristics of reported cytoskeletal protein in tapeworms.

Tapeworm	Protein	PM (kDa)	Isoforms	I. P.	Post-Translational Modifications
*T. crassiceps* [34]	Actin	41.99	7	5.14, 5.3, 5.49, 5.63, 5.79, and 5.9	Acetylation, methylation and ADP-ribosylation, ubiquitin, tyr.nitration, SUMOylation, phosphorylation, arginylation, Cys-oxidation [104]
*T. solium* [105]	Myosin II	250	1 (adult), 3 (cysticerci)	ND	Acetylation and phosphorylation [106]
*T. crassiceps* [34]	Paramyosin	102	3	4.8, 4.95, and 5.1	ND
*E. granulosus* [59]	Tropomyosin	32.26	3	4.6 **	Acetylation and phosphorylation [107,108]
*T. crassiceps* [34]	α-tubulin	51.92	4–5	4.7–5.7	Acetylation, Tyrosination, Ubiquitylation, Sumoylation, Phosphorylation, Palmitoylation, Glycososylation, Polyamination, Glycylation, Glutamylation [62]
*T. crassiceps* [34]	β-tubulin	51.92	4	4.5–5.2	Glutamylation, Glycylation, Glycososylation, Phosphorylatio [62]
*E. granulosus* [39]	Dynein	9.14	53 *	7.15	Phosphorylation [109]

ND: not determined, * Inferred value, ** Inferred value using the amino acid sequence.

**Table 2:** Isovariants of cytoskeleton genes identified in tapeworms.

Gene/ Parasite **	*E. g*	*E. m*	*H. d*	*H. n*	*M. c*	*T. a*	*T. s*	*T. c*
Actin	16	17	48 *	45 *	51 *	201 *	132 *	10
Dynein	73, 1 *	75, 1 *	28, 37 *	26, 45 *	27, 54 *	14, 99 *	1, 111 *	80
Gelsolin	2	2	1, 4 *	2, 4 *	2, 4 *	1, 5 *	7 *	3
Keratin	1	2	1 *	1 *	2 *	1 *	1 *	0
Kinesin	25	29	15, 9 *	16, 3 *	24, 5 *	9, 27 *	32 *	35
Myosin	12, 1 *	12, 1 *	7, 14 *	8, 9 *	5, 13 *	15 *	26 *	16
Nexin	7	7	1, 2 *	2, 2 *	1	5 *	8 *	15
Paramyosin	5	5	8	4	4	3	1	2
Tropomyosin	4	4	14 *	16 *	2, 13 *	14 *	5 *	5
Tubulin	28	29	22, 15 *	21, 17 *	23, 16 *	9, 37 *	62 *	38
Septin	4	4	4	4	4	2, 2 *	5 *	5
Vimentin	1	1	1 *	1 *	1 *	1 *	1 *	1

* Not-annotated gene. ** E. g: E. granulosus; E. m: E. multilocularis; H. d: H. diminuta; H. n: H. nana; M. c: M. corti, T. s: T. solium, T. c: T. crassiceps.

**Table 3:** Tapeworm cytoskeletal proteins as targets for drug treatment and vaccine and diagnostic tool development.

Protein	Species	Used for
Actin	*E. granulosus*	Diagnosis [130]
Paramyosin	*T. solium*, *E. granulosus*	Diagnosis/Vaccine development [52,130,137,140]
Tropomyosin	*T. solium*	Diagnosis/Vaccine development [127,141]
Tubulin	*T. solium*, *E. granulosus*	Diagnosis/Drug treatment [127,142]
Dynein	*A. perfoliata*	Diagnosis [136]
Severin	*E. granulosus*	Diagnosis [130]
Gelsolin	*E. granulosus*	Vaccine development/Drug treatment [141,143]

## Data Availability

Not applicable.
